# Single-Nucleotide Polymorphism Array-Based Karyotyping of Acute Promyelocytic Leukemia

**DOI:** 10.1371/journal.pone.0100245

**Published:** 2014-06-24

**Authors:** Inés Gómez-Seguí, Dolors Sánchez-Izquierdo, Eva Barragán, Esperanza Such, Irene Luna, María López-Pavía, Mariam Ibáñez, Eva Villamón, Carmen Alonso, Iván Martín, Marta Llop, Sandra Dolz, Óscar Fuster, Pau Montesinos, Carolina Cañigral, Blanca Boluda, Claudia Salazar, Jose Cervera, Miguel A. Sanz

**Affiliations:** 1 Hematology Department, Hospital Universitari i Politècnic La Fe, Valencia, Spain; 2 Array's Unit. Instituto Investigación Sanitaria Fundación La Fe, Valencia, Spain; 3 Laboratory of Molecular Biology, Department of Clinical Chemistry, University Hospital La Fe, Valencia, Spain; 4 Genetics Unit, Hospital Universitari i Politècnic La Fe, Valencia, Spain; 5 Department of Medicine, University of Valencia, Valencia, Spain; University of North Carolina School of Medicine, United States of America

## Abstract

Acute promyelocytic leukemia (APL) is characterized by the t(15;17)(q22;q21), but additional chromosomal abnormalities (ACA) and other rearrangements can contribute in the development of the whole leukemic phenotype. We hypothesized that some ACA not detected by conventional techniques may be informative of the onset of APL. We performed the high-resolution SNP array (SNP-A) 6.0 (Affymetrix) in 48 patients diagnosed with APL on matched diagnosis and remission sample. Forty-six abnormalities were found as an acquired event in 23 patients (48%): 22 duplications, 23 deletions and 1 Copy-Neutral Loss of Heterozygocity (CN-LOH), being a duplication of 8(q24) (23%) and a deletion of 7(q33-qter) (6%) the most frequent copy-number abnormalities (CNA). Four patients (8%) showed CNAs adjacent to the breakpoints of the translocation. We compared our results with other APL series and found that, except for dup(8q24) and del(7q33-qter), ACA were infrequent (≤3%) but most of them recurrent (70%). Interestingly, having CNA or *FLT3* mutation were mutually exclusive events. Neither the number of CNA, nor any specific CNA was associated significantly with prognosis. This study has delineated recurrent abnormalities in addition to t(15;17) that may act as secondary events and could explain leukemogenesis in up to 40% of APL cases with no ACA by conventional cytogenetics.

## Introduction

Acute promyelocytic leukemia (APL) is characterized by the t(15;17)(q22;q21) and the corresponding fusion gene *PML-RARA.* Additional chromosomal abnormalities (ACA) have been traditionally analyzed by conventional cytogenetics and fluorescence *in situ* hybridization (FISH). In the last decade, the single-nucleotide polymorphism array (SNP-A) has become a powerful tool to perform what has been called “molecular karyotyping”, because it increases the resolution of conventional cytogenetics and detects a wider spectrum of abnormalities than FISH and other targeted techniques. Moreover, SNP-A are able to uncover regions of copy-neutral loss of heterozygosity (CN-LOH), a known phenomenon that occurs in cancer and which is not detectable by conventional cytogenetics.[1]

Most reports studying ACA in APL with SNP-A have used low resolution arrays or have not systematically matched tumor and germline sample,[2], [3] which is the only certain way to rule out copy-number variations (CNV).[4] Our interest in the clinical impact of ACA in APL was addressed in previous studies,[5], [6] but these were performed with conventional cytogenetics and FISH. We hypothesize that there may be some ACA not detected by conventional techniques that could be detected by high resolution SNP-A karyotype.

In this report, we have performed SNP-A in a series of APL patients to learn the incidence of cryptic ACA, as well as to study any possible clinical or biologic association.

## Methods

### Ethics Statement

In accordance with the Declaration of Helsinki, this study was approved by the Research Ethics Board of our hospital (CEIB; *Comité Ético de Investigación Biomédica*). According to the Spanish law, written informed consent was obtained from all patients to participate in this study.

### Patients and samples

Patients consecutively diagnosed with APL between November 1998 and February 2011 in the Hospital Universitari i Politècnic La Fe with available tumor and germline DNA sample were selected for this study. DNA was provided by Biobank La Fe. Tumor DNA was obtained from bone marrow cells at diagnosis. Matched germline DNA was obtained from bone marrow or peripheral blood when qRT-PCR was negative for PML-RARA rearrangement. Conventional cytogenetics, FISH or RT-PCR for the detection of PML-RARA fusion were performed in every case, as well as tests for mutation detection of *FLT3-ITD* and D835 as previously described.[7] Patients were enrolled in three consecutive multicenter PETHEMA trials (LPA96, LPA99, and LPA2005).[8], [9] Clinical data as well as treatment outcome and follow-up were collected prospectively.

### SNP-A

Samples (500ng) were genotyped with Affymetrix GeneChip Human Mapping 6.0 according to manufacturer's protocol (Affymetrix Santa Clara, C.A., U.S.A.). DNA copy number and paired LOH analysis were performed using the Genotyping Console and the Chromosome Analysis Suite (ChAS) software (Affymetrix). Filters applied for the detection of segmental copy-number abnormalities (CNA) were ≥10 consecutive markers in a region of at least 10Kb, and for regions of CN-LOH, ≥50 markers in at least 100Kb. All abnormalities found in the remission sample were ruled out and assumed as non-somatic. In addition, every potential abnormality was checked in the Database of Genomic Variants (http://projects.tcag.ca/variation). Size, position, and location of genes were identified with UCSC Genome Browser (http://genome.ucsc.edu/). The human reference sequence used for alignment was the GRCh37/hg19assembly.

### SNP-A data from other APL series

We compared our results with other APL series, namely APL cases from The Cancer Genome Atlas (TCGA) Network with publicly available SNP-A data (n = 20), the series of Akagi *et al.* (n = 47) and the series of Nowak *et al.* (n = 93).[2], [3] Lesions found in the TCGA cohort are listed in Table S1 in Appendix S1. Lesions found in the series of Akagi *et al.* and Nowak *et al.* were listed in the corresponding report.[2], [3]

Conversion of hg18 to version hg19 was done in these cases using the “Batch Coordinate Conversion (liftOver)” Tool from the UCSC Genome Browser, with a minimum ratio of bases that remapped >0.95.

### Statistical analysis

Chi-square and Fisher's exact tests were used to analyze differences in the distribution of categorical variables. Mann–Whitney U-test was used to analyze differences in mean ranks. Unadjusted time-to-event analyses were performed using the Kaplan–Meier estimate and for comparisons, log-rank tests. Last update on clinical data was performed on March 2012. Median follow-up of patients alive was 77 months (range 13–151 mo.).

To investigate any possible association of the SNP-A abnormalities with clinical and biological characteristics, patients were divided according to the presence or not of CNA, the number of CNA and any recurrent CNA with n>3, such as +8/8q and CNA adjacent to the translocation breakpoints. Regarding the other APL series, we had available data for overall survival (OS) analysis from TCGA (n = 20) and Nowak *et al.* cohort (n = 89), and for relapse-free survival (RFS) from Nowak *et al.* cohort (n = 72 patients who achieved complete remission).

All computations were performed using the statistical package SPSS, version 17.0 (SPSS Inc., Chicago, IL, USA). A two-sided *P* value below .05 was considered significant.

## Results

### Identification of CNA and CN-LOH by SNP-A analysis

**Figure 1:**
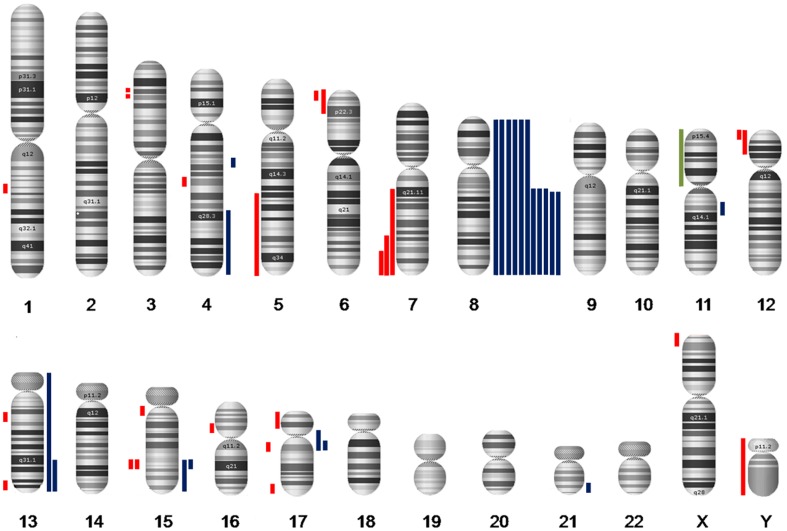
Karyogram of APL according to SNP-A analysis in our series. Coloured bars depict the extension of abnormalities. Gains appear in blue at the right of each chromosome; losses in red and CN-LOH in green, at the left side of it.

A total of 48 patients were included in this study. Main clinical and genetic characterization of our cohort is summarized in Table 1. Conventional cytogenetic studies were successful in 40 patients (83%). Seven of these patients (18%) had ACA (Table 1). SNP-A analysis revealed 46 abnormalities in 23 patients (48%). These consisted of 23 heterozygous deletions, 22 duplications and 1 CN-LOH (Figure 1). Ten (21%), 7 (15%) and 5 (12%) patients had one, two, and three or more anomalies, respectively.

**Table 1:** Main characteristics of our series.

Characteristics	Median (range)
Age	43 (17–76)
Leukocyte count (×10^9^/L)	2.2 (0–59)
Hemoglobin(g/dL)	9.0 (4.2–15.2)
Platelet count (x10^9^/L)	24.5 (2–168)
Bone marrow blasts (%)	90 (53–100)
	n (%)
Male gender	26 (54)
Therapy-related APL	3 (6)
Mophologic variant	
Classical	35 (73)
Hypogranular	13 (28)
Risk group (Sanz et al)^2^	
Low	9 (19)
Intermediate	26 (54)
High	13 (27)
Cytogenetics	
t(15;17)(q22;q21)	28 (58)
t(15;17)(q22;q21) and additional chromosomal abnormalities	7 (15)
Cryptic translocation*	5 (10)
Non-evaluable*	8 (17)
*PML-RARA* isoform type	
Bcr-1	28 (58)
Bcr- 2	2 (4)
Bcr- 3	18 (38)
*FLT3* mutational status	
*FLT3*-ITD	9 (19)
*FLT3*-D835	1 (2)
Treatment Protocol	
LPA-96	3 (6)
LPA-99	22 (46)
LPA-2005	23 (48)

*PML-RARA was diagnosed by FISH or RT-PCR.

A detailed list of the CNA and CN-LOH found in our series is shown in Table 2 and Figure 1. Median size of interstitial and telomeric CNA was 0.30 Mb (range, 0.11–19.38) and 77.48 Mb (range 3.45–146.36), respectively (Figure S1 in Appendix S1). No statistical difference in size was found for interstitial CNA (median size 0.46 Mb for duplications *vs.* 0.15 Mb for deletions).

**Table 2:** Detailed list of abnormalities found in our series (n = 48).

APL_ID	Copy Number State	Chr	Cytoband	Start Position	End Position	Size (Mb)
Duplication of chr. 8						
6	3	8	complete	0	146364022	146.36
23	3	8	complete	0	146364022	146.36
37	3	8	complete	0	146364022	146.36
1	1	6	p25.1–p24.3	4883499	7409514	2.53
	3	8	complete	0	146364022	146.36
11	3	8	q13.2-qter	68579063	146364022	77.48
	1	X	p22.33	0	3454962	3.45
26	3	8	complete	0	146364022	146.36
	3	11	q13.4	72283371	72418939	0.14
	1	12	p13.33	1027361	1134264	0.11
33	1	7	q21.11-qter	82589142	159119708	76.53
	3	4	q27-qter	123059206	191020138	67.96
	3	8	q12.3-qter	65285149	146364022	81.08
40	1	7	q31.32-qter	121167520	159138663	37.97
	3	8	q12.3-qter	65108706	146364022	81.26
46	1	5	q21.3-qter	107659808	180915260	73.26
	3	8	q12.3-qter	63759284	146364022	82.6
47	1	7	q33-qter	137199691	159138663	21.94
	3	8	q13.2-qter	68825854	146364022	77.54
**CNA in t(15;17) breakpoints**						
7	3	15	q24.1	74168174	74304138	0.14
20	1	15	q24.1	74691249	75117832	0.43
	1	17	q12	37959105	38079219	0.12
	3	21	q22.13–q22.2	39661746	39772776	0.11
32	1	17	pter-p11.2	0	19050914	19.05
	1	Y	complete	0	59373566	59.37
	3	15	q24.1-qter	74326556	102459244	28.13
	3	17	p11.2–q21.2	19093472	38477259	19.38
36	3	8	complete	0	146364022	146.36
	3	13	complete	0	115169878	115.17
	3	15	q24.1	74145053	74314995	0.17
	3	17	q21.2	38505591	38673928	0.17
**Others**						
3	1	16	p12.1	24507155	24661476	0.15
9	1	1	q24.2	167718498	168149489	0.43
10	1	4	q24	105743315	105897864	0.15
13	1	12	pter-p12.3	0	18489408	18.49
	3	13	q31.1-qter	85060629	115169878	30.11
21	1	17	q25.3	76335284	76796083	0.46
22	3	4	q21.3	87551324	87700668	149.34
25	1	6	p25.1-p22.3	5544745	15635655	10.09
	1	15	q11.2-q12	23641501	27343875	3.7
28	CN-LOH	11	p15.5-p12	198509	42278838	42.08
29	1	3	p24.2	24782029	28427029	3.65
	1	3	p22.3	34388000	35859690	1.47
	1	13	q14.11	40282116	40786721	0.5
	1	13	q33.2-q33.3	105672486	109302864	3.63
	1	13	q33.3	109671057	109798831	0.13

### Recurrent SNP-A lesions

**Figure 2:**
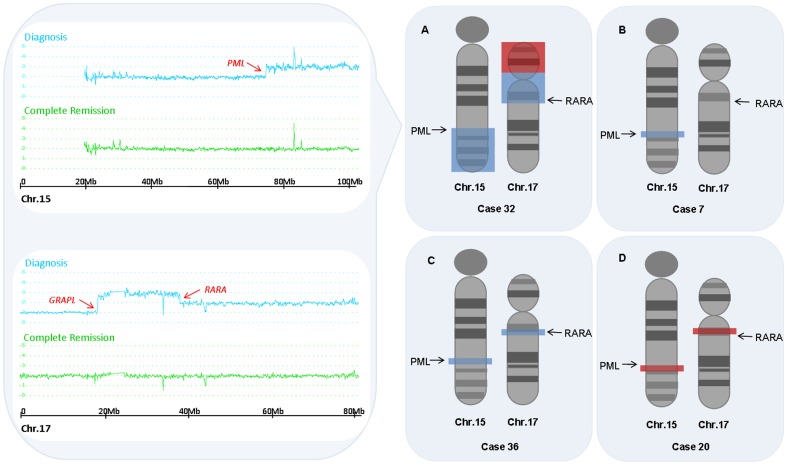
Schematic representation of CNA adjacent to the translocation breakpoints found in our series. On the left panel, the Smooth Signal of chromosome 15 and 17 from case APL_32 are represented. Results from diagnosis sample are shown in blue and from complete remission sample, in green. On the right, chromosomes 15 and 17 are depicted with G-banding and arrows pointing the location of the *PML* and *RARA* genes. Areas shaded in blue show regions duplicated and in red, deleted. Panel (A) corresponds to isochromosome der(17)t(15;17); Panel (B) shows a small duplication of the *PML* gene, and in panel (C) both the *PML* and the *RARA* genes are duplicated. These two cases had a cryptic t(15;17) by CC, that was revealed by FISH. In panel (D) two small deletions are found distally to the translocation breakpoints.

The most common abnormality was a duplication of +8/8q (11 patients, 23%), followed by a deletion of 7q (3 patients, 6%). Of note, 7 out of 11 +8/8q had not been detected by conventional cytogenetics. Four cases (8%) showed CNA adjacent to the breakpoints of the t(15;17) translocation (Figure 2). In case APL_20, two partial deletions were 0.5 Mb telomeric to *PML* and 0.5 Mb centromeric to *RARA*. In case APL_32, the SNP-A showed two large amplifications adjacent to the *PML* and the *RARA* gene and, additionally, a heterozygous partial deletion of 17p contiguous to the amplification. The breakpoint was in the coding region of *GRAPL* gene instead of the centromere. This event corresponds to an isochromosome of the derivative chromosome 17 of the translocation t(15;17) [ider(17)(q10)t(15;17)], which is occasionally seen in some APL cases by conventional cytogenetics. We could not confirm it in this case because no evaluable metaphases were obtained. In case APL_36, two small amplifications were found involving the *PML* and the *RARA* genes, and in case APL_7, a small amplification included part of the coding region of the *PML* gene. These 2 cases were among the 5 patients with cryptic translocation by conventional cytogenetics of our series.

**Figure 3:**
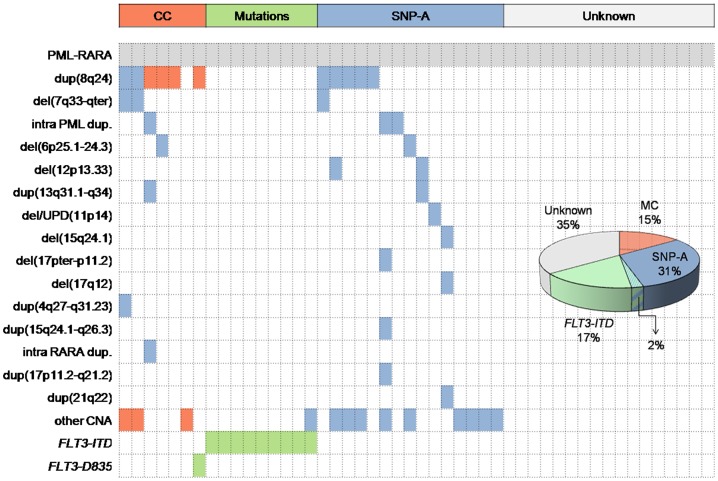
Heatmap and pie chart depicting abnormalities found in our series in addition to the t(15;17). Each column represents one patient. Abnormalities are listed in the Y axis and coloured in the corresponding row of the heatmap. Abnormalities detected by Conventional Cytogenetics (CC) (red) and *FLT3* mutations (green) are frequent events; however, SNP-A revealed an important proportion of patients with potentially leukemogeneic abnormalities (blue). Dup: duplication: del: deletion; CNA: copy-number abnormality.

Except for the 3 patients that had del(7q) and +8/8q concomitantly, no other association with clinical or biologic variables was observed. Conventional cytogenetics and *FLT3* mutation analysis revealed a secondary event to t(15;17) in 16 cases (33%). SNP-A uncovered cryptic additional abnormalities in 15 additional cases (31%). One third of cases still had no known secondary event (Figure 3). Patients carrying *FLT3-ITD* mutations had less frequently SNP-A anomalies (32% of mutations in patients without CNA *vs*. 4% in patients with at least one CNA; *P = *.016).

### Comparison with other series

The analysis of the type, size and number of lesions in each series is shown in Table 3. The series of Nowak *et al.* had fewer cases without CNA (17% in Nowak's series *vs*. 52%, 65% and 60% in our series, TCGA' and Akagi's series, respectively; *P*.001 in all three pairwise comparisons) and more abnormalities per case (median of 2 abnormalities in Nowak's series *vs*. 0 in all other report; *P*<.001 in all three pairwise comparisons). They also reported more deletions than duplications (65% of deletions in Nowak's series *vs*. 50% in this report and 29% in Akagi's series; *P* = .027 and .002, respectively) and a smaller size of each lesion (median size of 0.19 Mb *vs*. 18.77 Mb, 9.53 Mb and 45.11 Mb in this report, TCGA' and Akagi's series; *P*<.001,  = .018 and <.001, respectively). Akagi *et al.* reported more CN-LOH (21% of CN-LOH *vs.* 2% and 3% in this report and Nowak's series; *P* = .009 and <.001, respectively).

**Table 3:** Comparison of type, size and number of SNP-A abnormalities in reported APL series.

	Akagi *et al.*	TCGA	Nowak *et al.*	This Report
Number of patients	n = 47	n = 20	n = 93	n = 48
Array type	50 k & 250 K	SNP 6.0	SNP 6.0	SNP 6.0
Use of germline sample	7 (15%)	0 (0%)	3 (3%)	48 (100%)
Patient's abnormalities				
No CNA	28 (60%)	13 (65%)	16 (17%)	25 (52%)
Trisomy 8/8q+	8 (17%)	2 (10%)	25 (27%)	11 (23%)
Others	11 (23%)	5 (25%)	52 (56%)	12 (25%)
CNA types				
Duplications	17 (50%)	7 (54%)	83 (32%)	22 (48%)
Deletions	10 (29%)	6 (46%)	171 (65%)	23 (50%)
CN-LOH	7 (21%)	1 (5%)*	7 (3%)	1 (2%)
Total	34 (100%)	13 (100%)	261 (100%)	46 (100%)
CNA per case				
median	0	0	2	0
media	0.72	0.75	2.78	0.96
range	0–4	0–4	0–14	0–5
Size (Mb)				
median	45.11	9.53	0.19	18.77
media	55.06	25.65	16.38	41.72
range	0.02–146.36	0.13–146.36	0.001–191.15	0.11–146.36

*one Uniparental Tetrasomy has been included both in duplication and CN-LOH group.

CNA: Copy-Number Abnormality. CN-LOH: Copy-Neutral Loss of Heterozygocity.

The common deleted region (CDR) or the common gained region (CGR) of the CNA found in our series were delineated taking into account the boundaries reported by us and the other APL series (Table 4). Many CDR/CGR harbored well-known genes implicated in leukemia, such as *MYC*, *NF1*, *TP53* or *EZH2*. In every series, CGR 8(q24) was, by far, the most frequent abnormality (ranging from 10% to 25%), followed by CDR 7(q33-qter) (ranging from 2% to 6%). The remaining CDR/CGR were present in a lower frequency (≤3% of patients). One third (n = 68 out of 208) of the whole cohort carried at least one of the recurrent CDR/CGR. Most of the abnormalities found in our series (70%; n = 32) were recurrent in the other series, and the remaining CNA were known abnormalities in myeloid neoplasms, such as 5q- and –Y.

**Table 4:** Minimal deleted or gained regions found in APL series, their frequency and the genes included in each region.

Minimal deleted region (MDR)	Proximal	Distal	Size (Mb)	Akagi *et al.* (n = 47)	TCGA (n = 20)	Nowak et al. (n = 93)	This Report (n = 48)	Total n = 208	Genes
del(6p25.1–24.3)	5600438	7409514	1,81	1 (2%)	0	0	2 (4%)	3 (1%)	*FARS2, NRN1, F13A1, LY86, RREB1, CAGE1.*
del(6p24.1)	12278913	13386241	1,11	0	0	1 (1%)	1 (1%)	2 (1%)	*EDN1, PHACTR1, TBC1D7, GF0D1.*
del(7q33-qter)	137199691	158861884	21,66	1 (2%)	1 (5%)	5 (5%)	3 (6%)	10 (5%)	several genes including *LUC7L2, EZH2.*
del/UPD(11p14)	22936456	30671304	7,73	3 (6%)	1 (5%)	0	1 (1%)	5 (2%)	*LUZP2, ANO3, MUC15, SLC5A12, BBPX1, CCDC34, LGR4, LIN7C, BDNF, METTL15, KCNA4, FSHB, MPPED2.*
del(12p13.33)	1027361	1134264	0,11	0	0	0	2 (4%)	2 (1%)	*RAD52, ERC1.*
del(15q24.1)	74691249	75117832	0,43	0	0	1 (1%)	1 (1%)	2 (1%)	*SEMA7A, UBL7, ARID3B, CLK3, EDC3, CYP1A1, CSK, LMAN1L.*
del(17pter-p11.2)	0	18935523	18,94	1 (2%)	0	3 (3%)	1 (1%)	5 (2%)	several genes including *TP53.*
del(17q12)	37959105	38079219	0,12	0	0	1 (1%)	1 (1%)	2 (1%)	*IKZF3, GSDMB, ZPBP2, ORMDL3.*
**Minimal gained region (MGR)**									
dup(4q27–q31.23)	123059206	150420462	27,36	0	0	5 (5%)	1 (1%)	6 (3%)	several genes.
dup(8q24)	122538604	132023578	9,48	8 (17%)	2 (10%)	23 (25%)	11 (23%)	44 (21%)	several genes including MYC.
dup(13q31.1–q34)	85060629	115108397	30,05	1 (2%)	0	1 (1%)	2 (4%)	4 (2%)	several genes.
dup(15q24.1) intra *PML*	74262253	74304138	0,04	0	0	4 (4%)	2 (4%)	6 (3%)	*PML.*
dup(15q24.1–q26.3)	74326556	102364660	28,04	2 (4%)	0	3 (3%)	1 (1%)	6 (3%)	several genes including *PML.*
dup(17q21.2) intra *RARA*	38505591	38673928	0,17	0	0	1 (1%)	1 (1%)	2 (1%)	*RARA.*
dup(17p11.2–q21.2)	21550542	38477259	16,93	1 (2%)	0	3 (3%)	1 (1%)	5 (2%)	several genes including *NF1, RARA*
dup(21q22)	39661746	39772776	0,11	2 (4%)	0	1 (1%)	1 (1%)	5 (2%)	*KCNJ15, ERC.*

Two patterns of CNAs adjacent to the t(15;17) breakpoints were found recurrently. The first one was the pattern corresponding to ider(17)(q10)t(15;17) (5 cases, 2%). The breakpoint leading to deletion of 17p was found within the coding region of the *GRAPL* gene, the *GRAP* gene, or even more centromeric. The second recurrent pattern was the duplication of the *PML* locus without reciprocal duplication of the *RARA* locus, (5 cases, 2%).

We observed again that CGR 8(q24) and CDR 7(q33-qter) appeared concomitantly [80% of deletions in 7(q33-qter) in patients with dup(8q24) *vs.* 18% in patients without dup(8q24); P <.001].

### Correlation with clinical data

No single CNA showed an association with clinical variables or a prognostic impact in terms of OS and RFS, as was also the case of those cases with cryptic CNA that were not observed by conventional cytogenetics, and those with 0, 1 or ≥2 CNAs revealed by SNP-A.

We analyzed next every recurrent CDR/CGR in the series with available survival data. All three series had independent similar OS and RFS. No recurrent CDR/CGR showed a prognostic value; however, patients who carried ≥1 recurrent CDR/CGR showed a shorter 3-year OS (86% *vs.* 75%; P = .05), and RFS (90% *vs.* 78% in patients having 0 *vs.* ≥1 recurrent lesion; P = .05) (Figure S2 in Appendix S1).

## Discussion

This study shows that high-resolution SNP-A analysis reveals ACA in roughly half the patients with APL (48%). Most of them (90%) had not been properly detected by conventional cytogenetics. Systematic use of matched diagnosis and molecular remission sample in every single case has allowed us to ascertain the somatic nature of these lesions. Moreover, the majority of them were recurrent (70%) but of low frequency (≤3%), and accounted for a secondary event in up to 40% of APL cases with no ACA by conventional cytogenetics.

Our analysis showed a low burden of CNA in our series, which is in line with previous reports of core-binding factor acute myeloid leukemia (AML),[10] and even lower than AML of any kind,[11] suggesting that few additional lesions are needed in this type of leukemias. These findings differ widely from the results reported by Nowak *et al.*,[3] where they found three times more lesions per case and with a much smaller size. It could be possible that these differences are due to the lack of systematic use of germline sample and assumption of some CNV as somatic when not reported in public CNV databases.

Among the SNP-A abnormalities, +8/8q was the most frequent in our series and the other reports.[2], [3] Trisomy 8 is known to be the most frequent chromosomal abnormality in APL [5] and very common in myeloid neoplasms. However, SNP-A analysis allowed for a three-fold detection of duplications in chromosome 8 in our series. Of note, the CGR had a size of 9.5Mb and comprised few genes, including *MYC* as the most probable candidate gene, which has been shown to be deregulated and amplified in several types of leukemias and lymphomas.[12], [13] The second most recurrent abnormality was del(7q), which appeared in association with dup(8q24), both common CNA in myeloid neoplasms. Regarding CN-LOH, most series of APL and other translocation-associated leukemias have reported a very low rate (<5%),[3], [10], [14] which is in line with our findings and suggest a very low frequency of this event in APL.

CNA in the boundaries of t(15;17) breakpoints were disclosed in some patients. This also occurs in other supposedly balanced translocations in an even higher frequency (around 25% of core binding factor AML and 10% in CML).[10], [14] Two patterns seemed to be recurrent: the first one corresponded to an isochromosome derivative of chromosome 17(t15;17). Isochromosome 17q [i(17q)] is not monocentric but a dicentric chromosome. It has a breakpoint cluster region located at 17p11.2, which is a genetically unstable region that contains multiple low-copy repeats and segmental duplications.[15] We located the breakpoint region at the *GRAPL* gene. Other authors have located the breakpoint at the *GRAP* gene [3] or more centromeric in a non-coding region,[2] but all of them are within this breakpoint cluster region. This region has shown susceptibility to rearrangement and explains the relative high frequency of i(17q) in hematologic and non-hematologic cancers, as well as deletions in this area in Smith-Magenis syndrome.[15], [16] The fact that the i(17q) harbours the t(15;17) indicates that the i(17q) occurs as a secondary event, that in addition will produce an extra RARA-PML transcript.[17]

Cases with duplication of the *PML* gene were recurrent, and also similar cases have been reported before.[18] It is interesting that some of these cases had a cryptic t(15;17) according to conventional cytogenetics. APL cases with cryptic t(15;17) may be due to small interstitial insertions of *PML* or *RARA* genes one beside the other [19] or even ectopic to the natural gene loci. [18], [20], [21] Small deletions very close to the translocated genes have been reported in 10-30% of patients with CBF-AML [t(8;21) and inv(16)] and CML [t(9;22)], usually involving the translocated genes.[10], [14] Less frequently (∼1%), some cases have been described in such leukemias with deletions located in more telomeric or centromeric regions from the translocated gene, [10], [14] resembling our case APL_20. The study of such cases with imbalanced translocations may help to elucidate the mechanisms that give rise to the t(15;17).

The parallel study of *FLT3-ITD* mutations and CNA by SNP-A showed a mutually exclusive association of these events. This is in agreement with previous studies [2] and supports the hypothesis that tyrosin kinase activation through *FLT3-ITD* mutations is a sufficient cooperating event to produce APL and therefore, other genomic aberrations such as CNAs are not needed. In our series, *FLT3* mutations and conventional cytogenetics revealed an additional abnormality in one third of patients. Interestingly, SNP-A analysis uncovered one additional third of patients from this cohort that carried acquired CNA/CN-LOH in the leukemic blasts. Most of those abnormalities were infrequent (≤3% of patients) but recurrent, indicating a role of these lesions in the development of APL and therefore the capability of high-resolution SNP-A karyotype to reveal hidden leukemogenic events. The majority of these recurrent CNAs and also some non-recurrent are well-known abnormalities in myeloid neoplasms. This suggests that additional events which occur after the t(15;17) are not necessarily exclusive of APL, but common with acute myeloid leukemia and other hematologic neoplasms. Likewise, these facts highlight the wide degree of heterogeneity seen in the cooperating events that contribute to APL.

Still another third of patients had neither *FLT3* mutations nor ACA. Probably, more advanced technologies such as next-generation sequencing will help to elucidate the abnormalities acquired in these cases.

No independent association with prognosis could be established for any recurrent CNA, although we are aware of the small size of our series for this type of analysis. Even the most frequent CNA, dup(8q24), that has been assigned a prognostic value in certain hematologic neoplasms,[22], [23] was not related to the patient's outcome, as was also the case in previous reports of APL.[3], [5] However, patients who lacked an available remission sample, such as those who died before achieving complete remission, were not included in this study. This last fact may constitute a selection bias.

Carrying one or more CDR/CGR showed a trend towards a worse prognosis, although our cohort was heterogeneous for survival analysis and besides, multivariate analysis could not be performed. More solid evidence will therefore be required to confirm this finding. However, it is true that similar results have been reported before in other hematologic neoplasms [24]-[26] revealing that the more lesions in the cell's genome, the more complexity and poorer prognosis the disease has.

In summary, these data demonstrate an acquired lesion in leukemic blasts in 40% of patients with no ACA by conventional cytogenetics. The low frequency and recurrence of these ACA indicate the broad spectrum of abnormalities that can occur after the t(15;17) and are required for the leukemic transformation. Whether this picture can be fully explained using more comprehensive technology remains to be seen.

## Supporting Information

Appendix S1 **Supporting Information file.** Table S1. Lesions found in the 20 APL cases of the TCGA cohort. Figure S1. Graphic representation of the size and type of Copy Number Abnormalities (CNA) in our series. Figure S2. Survival curves of the reported APL series according to the number of CDR/CGR.
(DOC)

## References

[1] O'Keefe C, McDevitt MA, Maciejewski JP (2010) Copy neutral loss of heterozygosity: a novel chromosomal lesion in myeloid malignancies. Blood 115: 2731–2739. 20107230 10.1182/blood-2009-10-201848 PMC2854422

[2] Akagi T, Shih LY, Kato M, Kawamata N, Yamamoto G, et al (2009) Hidden abnormalities and novel classification of t(15;17) acute promyelocytic leukemia (APL) based on genomic alterations. Blood 113: 1741–1748. 19109227 10.1182/blood-2007-12-130260 PMC2647673

[3] Nowak D, Klaumuenzer M, Hanfstein B, Mossner M, Nolte F, et al (2012) SNP array analysis of acute promyelocytic leukemia may be of prognostic relevance and identifies a potential high risk group with recurrent deletions on chromosomal subband 1q31.3. Genes Chromosomes Cancer 51: 756–767. 22488577 10.1002/gcc.21961

[4] Heinrichs S, Li C, Look AT (2010) SNP array analysis in hematologic malignancies: avoiding false discoveries. Blood 115: 4157–4161. 20304806 10.1182/blood-2009-11-203182 PMC2879098

[5] Cervera J, Montesinos P, Hernandez-Rivas JM, Calasanz MJ, Aventin A, et al (2010) Additional chromosome abnormalities in patients with acute promyelocytic leukemia treated with all-trans retinoic acid and chemotherapy. Haematologica 95: 424–431. 19903674 10.3324/haematol.2009.013243 PMC2833072

[6] Hernandez JM, Martin G, Gutierrez NC, Cervera J, Ferro MT, et al (2001) Additional cytogenetic changes do not influence the outcome of patients with newly diagnosed acute promyelocytic leukemia treated with an ATRA plus anthracyclin based protocol. A report of the Spanish group PETHEMA. Haematologica 86: 807–813. 11522536

[7] Barragan E, Montesinos P, Camos M, Gonzalez M, Calasanz MJ, et al (2011) Prognostic value of FLT3 mutations in patients with acute promyelocytic leukemia treated with all-trans retinoic acid and anthracycline monochemotherapy. Haematologica 96: 1470–1477. 21685470 10.3324/haematol.2011.044933 PMC3186308

[8] Sanz MA, Martín G, González M, León A, Rayón C, et al (2004) Risk-adapted treatment of acute promyelocytic leukemia with all-trans-retinoic acid and anthracycline monochemotherapy: a multicenter study by the PETHEMA group. Blood 103: 1237–1243. 14576047 10.1182/blood-2003-07-2462

[9] Sanz MA, Montesinos P, Rayón C, Holowiecka A, de la Serna J, et al (2010) Risk-adapted treatment of acute promyelocytic leukemia based on all-trans retinoic acid and anthracycline with addition of cytarabine in consolidation therapy for high-risk patients: further improvements in treatment outcome. Blood 115: 5137–5146. 20393132 10.1182/blood-2010-01-266007

[10] Kuhn MW, Radtke I, Bullinger L, Goorha S, Cheng J, et al (2012) High-resolution genomic profiling of adult and pediatric core-binding factor acute myeloid leukemia reveals new recurrent genomic alterations. Blood 119: e67–75. 22234698 10.1182/blood-2011-09-380444 PMC3311263

[11] Walter MJ, Payton JE, Ries RE, Shannon WD, Deshmukh H, et al (2009) Acquired copy number alterations in adult acute myeloid leukemia genomes. Proc Natl Acad Sci U S A 106: 12950–12955. 19651600 10.1073/pnas.0903091106 PMC2716381

[12] Delgado MD, Albajar M, Gomez-Casares MT, Batlle A, Leon J (2013) MYC oncogene in myeloid neoplasias. Clin Transl Oncol 15: 87–94. 22911553 10.1007/s12094-012-0926-8

[13] Slack GW, Gascoyne RD (2011) MYC and aggressive B-cell lymphomas. Adv Anat Pathol 18: 219–228. 21490439 10.1097/PAP.0b013e3182169948

[14] Huh J, Jung CW, Kim JW, Kim HJ, Kim SH, et al (2011) Genome-wide high density single-nucleotide polymorphism array-based karyotyping improves detection of clonal aberrations including der(9) deletion, but does not predict treatment outcomes after imatinib therapy in chronic myeloid leukemia. Ann Hematol 90: 1255–1264. 21384125 10.1007/s00277-011-1195-2

[15] Barbouti A, Stankiewicz P, Nusbaum C, Cuomo C, Cook A, et al (2004) The breakpoint region of the most common isochromosome, i(17q), in human neoplasia is characterized by a complex genomic architecture with large, palindromic, low-copy repeats. Am J Hum Genet 74: 1–10. 14666446 10.1086/380648 PMC1181896

[16] Chen KS, Manian P, Koeuth T, Potocki L, Zhao Q, et al (1997) Homologous recombination of a flanking repeat gene cluster is a mechanism for a common contiguous gene deletion syndrome. Nat Genet 17: 154–163. 9326934 10.1038/ng1097-154

[17] Manola KN, Karakosta M, Sambani C, Terzoudi G, Pagoni M, et al (2010) Isochromosome der(17)(q10)t(15;17) in acute promyelocytic leukemia resulting in an additional copy of the RARA-PML fusion gene: report of \ases and review of the literature. Acta Haematol 123: 162–170. 20224268 10.1159/000294959

[18] Koshy J, Qian YW, Bhagwath G, Willis M, Kelley TW, et al (2012) Microarray, gene sequencing, and reverse transcriptase-polymerase chain reaction analyses of a cryptic PML-RARA translocation. Cancer Genet 205: 537–540. 22982005 10.1016/j.cancergen.2012.07.017

[19] Goldschmidt N, Yehuda-Gafni O, Abeliovich D, Slyusarevsky E, Rund D (2010) Interstitial insertion of RARalpha gene into PML gene in a patient with acute promyelocytic leukemia (APL) lacking the classic t(15;17). Hematology 15: 332–337. 20863428 10.1179/102453310X12647083621083

[20] Haraguchi K, Ohno N, Tokunaga M, Itoyama T, Gotoh M, et al (2009) Masked t(15;17) APL with the insertion of PML-RARalpha fusion gene in 4q21. Leuk Res 33: 1552–1555. 19477514 10.1016/j.leukres.2009.04.033

[21] Garcia-Casado Z, Cervera J, Valencia A, Pajuelo JC, Mena-Duran AV, et al (2006) A t(17;20)(q21;q12) masking a variant t(15;17)(q22;q21) in a patient with acute promyelocytic leukemia. Cancer Genet Cytogenet 168: 73–76. 16772124 10.1016/j.cancergencyto.2005.12.014

[22] Such E, Cervera J, Costa D, Solé F, Vallespí T, et al (2011) Cytogenetic risk stratification in chronic myelomonocytic leukemia. Haematologica 96: 375–383. 21109693 10.3324/haematol.2010.030957 PMC3046268

[23] Greenberg PL, Tuechler H, Schanz J, Sanz G, Garcia-Manero G, et al (2012) Revised International Prognostic Scoring System for Myelodysplastic Syndromes. Blood 120: 2454–2465. 22740453 10.1182/blood-2012-03-420489 PMC4425443

[24] Parkin B, Erba H, Ouillette P, Roulston D, Purkayastha A, et al (2010) Acquired genomic copy number aberrations and survival in adult acute myelogenous leukemia. Blood 116: 4958–4967. 20729466 10.1182/blood-2010-01-266999 PMC3012590

[25] Tiu RV, Gondek LP, O'Keefe CL, Huh J, Sekeres MA, et al (2009) New lesions detected by single nucleotide polymorphism array-based chromosomal analysis have important clinical impact in acute myeloid leukemia. J Clin Oncol 27: 5219–5226. 19770377 10.1200/JCO.2009.21.9840 PMC2773477

[26] Schweighofer CD, Coombes KR, Majewski T, Barron LL, Lerner S, et al (2013) Genomic Variation by Whole-Genome SNP Mapping Arrays Predicts Time-to-Event Outcome in Patients with Chronic Lymphocytic Leukemia: A Comparison of CLL and HapMap Genotypes. The Journal of molecular diagnostics: JMD 15: 196–209. 23273604 10.1016/j.jmoldx.2012.09.006 PMC3586684

