# Supplementary material for: Single-Nucleotide Polymorphism Array-Based Karyotyping of Acute Promyelocytic Leukemia
Source: PLoS One. 2014 Jun 24;9(6):e100245. doi: 10.1371/journal.pone.0100245 (PMC4069034; doi:10.1371/journal.pone.0100245)

**Single-nucleotide Polymorphism Array-based Karyotyping of Acute**

**Promyelocytic Leukemia**

**SUPPLEMENTARY APPENDIX**

*Inés Gómez-Seguí,^1^ Dolors Sánchez-Izquierdo,^2^ Eva Barragán,^3^ Esperanza Such,^1^ Irene Luna,^1^ María López-Pavía, ^1^ Mariam Ibáñez, ^1^ Eva Villamón, ^1^ Carmen Alonso, ^1^ Iván Martín, ^1^ Marta Llop, ^3^ Sandra Dolz, ^3^ Óscar Fuster, ^3^ Pau Montesinos, ^1^ Carolina Cañigral, ^1^ Blanca Boluda, ^1^ Claudia Salazar, ^1^ Jose Cervera^1,4^ * and Miguel A. Sanz.^1,5^ **

**Both senior authors contributed equally to this work.*

**Table S1.** Lesions found in the 20 APL cases of the TCGA cohort. Chr: chromosome, UPT: uniparental tetrasomy.

| Sample | Lesion | Chr | Size (Kb) | Proximal | Distal |
| --- | --- | --- | --- | --- | --- |
| TCGA-2823 | Gain | 8p12 | 578,43 | 36158945 | 36737377 |
|  | Gain | 8p11.21 | 806,45 | 40084213 | 40890658 |
|  | Gain | 8p11.21q21.1 | 34391,86 | 43162524 | 77554388 |
|  | UPT | 8q23.2q24.3 | 35154,87 | 111114079 | 146268947 |
| TCGA-2840 | Gain | 10q25.1 | 331,71 | 109566288 | 109897996 |
| TCGA-2872 | Loss | 11p14.3p12 | 19160,3 | 22893032 | 42053336 |
|  | Loss | 9q12q31.1 | 35566,65 | 69267830 | 104834483 |
| TCGA-2905 | Loss | 4p15.31 | 131,76 | 21171470 | 21303229 |
| TCGA-2997 | Loss | 4q22.3 | 170,75 | 98207359 | 98378111 |
| TCGA-3007 | Gain | 8 | complete | - | - |
|  | Gain | 11q24.2q25 | 9529,07 | 124920917 | 134449982 |
|  | Loss | 7q31.1q36.3 | 51162,72 | 107617096 | 158779815 |
| TCGA-3012 | Loss | 11q22.1 | 159,94 | 98226669 | 98386612 |
| TCGA-2803 | no abnormality | | | | |
| TCGA-2804 | no abnormality | | | | |
| TCGA-2841 | no abnormality | | | | |
| TCGA-2862 | no abnormality | | | | |
| TCGA-2897 | no abnormality | | | | |
| TCGA-2906 | no abnormality | | | | |
| TCGA-2980 | no abnormality | | | | |
| TCGA-2982 | no abnormality | | | | |
| TCGA-2991 | no abnormality | | | | |
| TCGA-2994 | no abnormality | | | | |
| TCGA-2998 | no abnormality | | | | |
| TCGA-2999 | no abnormality | | | | |
| TCGA-3001 | no abnormality | | | | |

**Figure S1.** Graphic representation of the size and type of Copy Number Abnormalities (CNA) in our series. CNA can be divided in two groups according to its size and location: big telomeric CNA and small intersticial CNA.


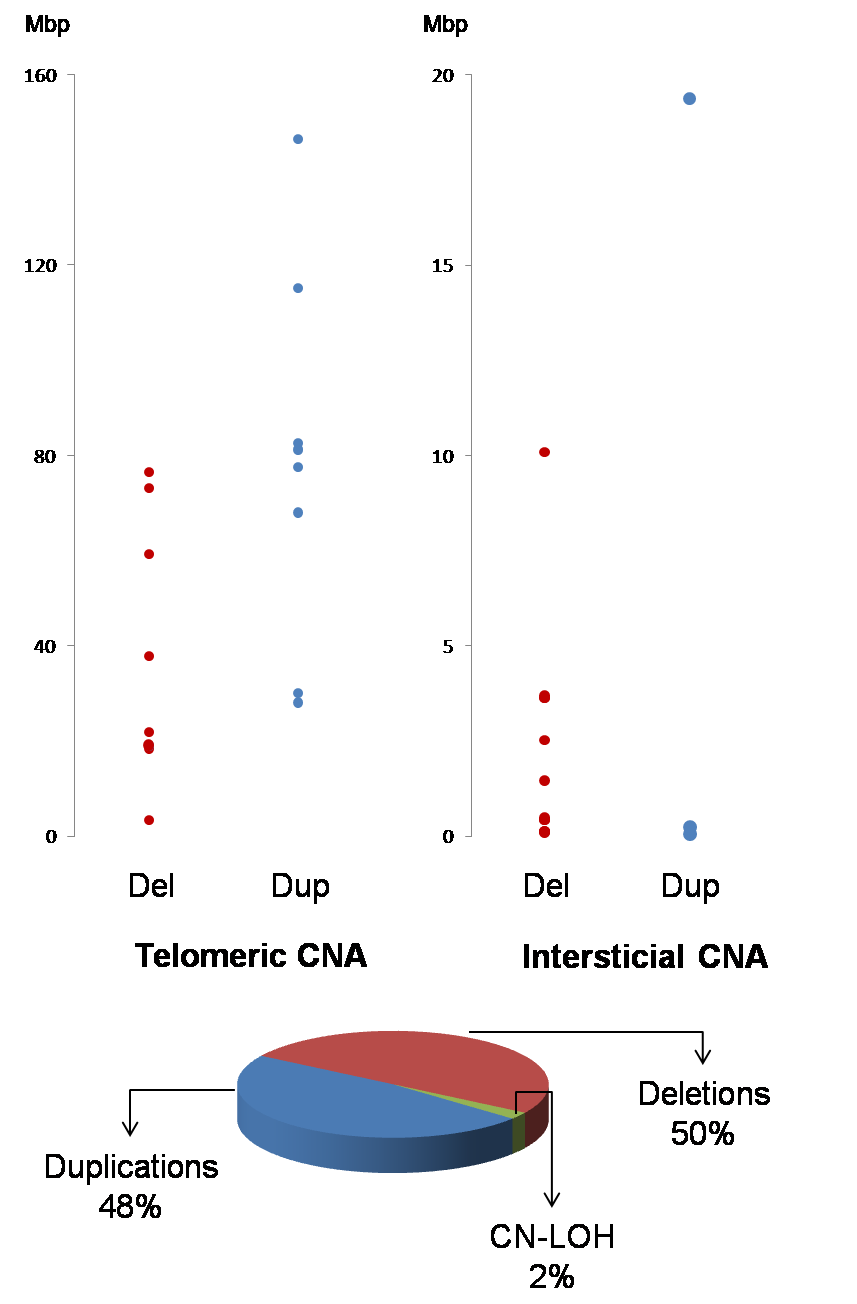


**Figure S2**. Survival curves of the reported APL series according to the number of CDR/CGR .


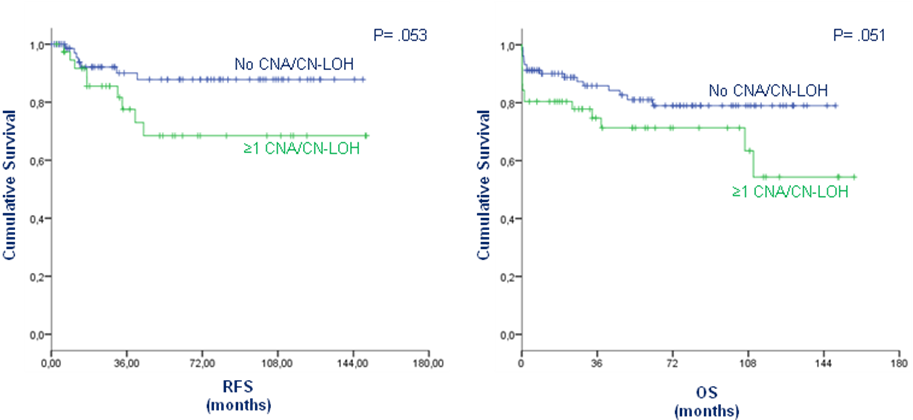

Supplement: Appendix S1 — Supporting Information file. Table S1. Lesions found in the 20 APL cases of the TCGA cohort. Figure S1. Graphic representation of the size and type of Copy Number Abnormalities (CNA) in our series. Figure S2. Survival curves of the reported APL series according to the number of CDR/CGR. (DOC) [file pone.0100245.s001.doc]
